# Targeting Atrial Fibrillation in HFpEF: Comparative Effectiveness of Rhythm Control Strategies and Modulatory Effects of SGLT2 Inhibitors

**DOI:** 10.3390/jcm14228003

**Published:** 2025-11-11

**Authors:** Marius-Dragoș Mihăilă, Ioan-Alexandru Minciună, Bogdan Caloian, Florina Iulia Frîngu, Samuel Bogdan Todor, Andrei Iulian Aleman, Dana Pop

**Affiliations:** 1 4th Department of Internal Medicine, Department of Cardiology Rehabilitation, “Iuliu Hațieganu” University of Medicine and Pharmacy, 400012 Cluj-Napoca, Romania; marius.drag.mihaila@elearn.umfcluj.ro (M.-D.M.); bogdan912@elearn.umfcluj.ro (B.C.); florina.fringu@elearn.umfcluj.ro (F.I.F.); pop67dana@gmail.com (D.P.); 2 Department of Cardiology Rehabilitation, Clinical Rehabilitation Hospital, 400437 Cluj-Napoca, Romania; andrei.iuli.aleman@elearn.umfcluj.ro; 3 Department of Haematology, Faculty of Medicine, “Lucian Blaga” University of Sibiu, 550169 Sibiu, Romania; samuelbogdant@gmail.com

**Keywords:** heart failure with preserved ejection fraction, atrial fibrillation, catheter ablation, electrical cardioversion, sodium–glucose cotransporter 2 inhibitors

## Abstract

**Background**: Atrial fibrillation (AF) is a common comorbidity in heart failure with preserved ejection fraction (HFpEF), yet the optimal rhythm control strategies and the emerging role of sodium–glucose cotransporter-2 inhibitors (SGLT2i) in maintaining sinus rhythm remain unclear. **Methods**: We conducted a single-centre, retrospective study of 120 consecutive HFpEF patients with paroxysmal or persistent AF, treated by electrical cardioversion, radiofrequency ablation (RFA), or cryoablation. The primary outcome was AF recurrence, with secondary outcomes including time to recurrence and the impact of SGLT2i on AF recurrence. **Results**: Both cryoablation (HR = 0.226, 95% CI: 0.089–0.573, *p* = 0.002) and RFA (HR = 0.293, 95% CI: 0.131–0.654, *p* = 0.003) were associated with lower AF recurrence rates and longer arrhythmia-free survival compared to electrical cardioversion. SGLT2i therapy was independently associated with fewer recurrences (HR = 0.421, 95% CI: 0.266–0.786, *p* = 0.007) and a 12-week extension of AF-free time. **Conclusions**: In HFpEF with AF, prioritising catheter ablation over cardioversion and combining rhythm control with SGLT2i improves rhythm durability.

## 1. Introduction

Heart failure with preserved ejection fraction (HFpEF) constitutes a distinct category of heart failure (HF) due to the numerous cardiovascular and non-cardiovascular comorbidities with which it is associated and which substantially influence its progression and prognosis [1]. The European Society of Cardiology (ESC) guidelines [2] also highlight proactive screening and management of comorbidities as a key aspect of care, alongside SGLT2i therapy, in HFpEF patients. Given the particular importance of the comorbidity profile in patients with HFpEF, their treatment must be approached in an individualised manner according to their different comorbid phenotypes [3].

Atrial fibrillation (AF) is one of the most common comorbidities in HFpEF and contributes to poorer clinical outcomes of treatment in this type of HF [4]. Invasive hemodynamic studies have confirmed that patients with AF are at higher risk of HFpEF, and that the presence of HFpEF may have important prognostic implications in these patients [5]. The coexistence of AF and HFpEF is also supported by the presence of several common risk factors: age, body mass index, arterial hypertension, diabetes mellitus, and obstructive sleep apnoea have been identified as comorbidities frequently associated with both conditions [6]. Systemic inflammation, hemodynamic alterations, microvascular dysfunction, epicardial adiposity, and myocardial fibrosis are all key consequences of the common risk factors outlined above and play a crucial role in the development of atrial myopathy underlying AF and HFpEF [7]. Besides these mechanisms, AF and HFpEF worsen each other’s development and progression, creating a vicious cycle that, if untreated, leads to faster deterioration of both conditions.

Regarding AF management, while catheter ablation has proven to improve prognosis in patients with heart failure with reduced ejection fraction (HFrEF), the benefits of AF ablation remain unclear in patients with HFpEF [8]. Recent research indicates a possible positive impact of AF ablation on adverse clinical outcomes, AF recurrence, and functional status in patients with a high likelihood of HFpEF, but further studies are needed to better understand its role in HFpEF management [9]. Also, given the accumulating evidence for the cardiovascular benefits of SGLT2i, and considering the variety of molecular targets and intracellular mechanisms through which they act, the question has recently been raised whether they might also exert protective antiarrhythmic effects in AF [10]. This is suggested by evidence that they appear to reduce the risk of AF recurrence, particularly after catheter ablation, thereby decreasing the need for antiarrhythmic drugs, electrical cardioversion, or re-do ablations [11].

Considering these aspects, we aimed to investigate the efficacy of different rhythm control strategies in patients with AF and HFpEF, namely electrical cardioversion, radiofrequency ablation (RFA), and cryoablation, as well as the extent to which treatment with SGLT2i could influence the outcomes of rhythm control strategies in these patients.

## 2. Materials and Methods

This retrospective observational cohort study was conducted in the Cardiology Department of the Cluj-Napoca Clinical Rehabilitation Hospital and involved 120 consecutive patients diagnosed with HFpEF and paroxysmal or persistent AF who underwent one of the following rhythm control strategies between January 2023 and October 2024: electrical cardioversion, RFA, or cryoablation. Patients who underwent multiple electrical cardioversions, re-do ablations, or for whom no data on AF recurrence were recorded were excluded from the study.

For each patient included in the study, data were extracted from the hospital’s electronic medical record system and the electrophysiology laboratory database: demographic information (age, sex), AF type (paroxysmal or persistent), the rhythm control method used, the prescribed antiarrhythmic medication after sinus rhythm restoration, the technical parameters of RFA and cryoablation procedures (radiation exposure dose, radiation exposure time, procedure duration), the presence or absence of AF recurrence, the time to AF recurrence in patients where it occurred, echocardiographic and biological parameters, and the presence or absence of treatment with an SGLT2i after sinus rhythm restoration. Additionally, information was collected regarding other cardiac and non-cardiac comorbidities present in these patients, along with details of drug treatments. All patient data were anonymised before analysis.

In all cases, the diagnosis of HFpEF was confirmed according to the current ESC criteria. In this retrospective cohort, AF recurrence was assessed at 3 months post-cardioversion/ablation (blanking period) using 12-lead ECG and/or Holter ECG monitoring as clinically indicated. Afterwards, follow-up was symptom-driven: patients were re-evaluated at the onset of palpitations or related symptoms to assess for AF recurrence. AF recurrence was defined as any episode of AF lasting ≥ 30 s, documented by ECG or Holter ECG monitoring, occurring beyond the 3-month post-ablation blanking period. Episodes within the first 3 months were classified as early recurrences and were not considered for this analysis.

The patients enrolled in the study were subsequently categorised into distinct groups based on the rhythm control strategy used and whether they received treatment with SGLT2i. The primary outcome was the recurrence of AF during the follow-up period. Secondary outcomes included the time to AF recurrence and the relationship between SGLT2i treatment and the maintenance of sinus rhythm.

The study was conducted in accordance with the regulations of the Declaration of Helsinki and received approval from both the Ethics Committee of the “Iuliu Hațieganu” University of Medicine and Pharmacy Cluj-Napoca (approval number AVZ155/30.07.2024) and the Clinical Rehabilitation Hospital Cluj-Napoca (approval number 9/01.03.2024).

### Statistical Analysis

Categorical variables were presented as numbers and percentages (*n*, %) and compared using the Chi-square test for independence. Continuous variables were displayed as medians and interquartile ranges (25–75 percentiles), and comparisons between groups were conducted using the Mann–Whitney U test or the Kruskal–Wallis test, depending on the number of groups analysed. Multiple linear regression was used to identify predictors associated with the time to AF recurrence. Survival analysis was conducted using Kaplan–Meier curves, and differences in survival between groups were evaluated with the log-rank test. Cox regression was used to identify independent predictors, with results expressed as hazard ratios (HRs) with 95% confidence intervals (95% CIs). Subsequently, to evaluate the predictive power of the multivariable model, the hazard probability was calculated for each case, and the obtained values were entered into a ROC analysis. The area under the curve (AUC) of the hazard probability estimated by the multivariable model served as an indicator of its predictive performance. All statistical tests were two-sided, and the level of statistical significance was set at α = 0.05. Statistical analyses were conducted using IBM SPSS Statistics, version 24.0 (IBM Corporation, Armonk, New York, NY, USA).

## 3. Results

In this retrospective study, we examined the risk of AF recurrence in 120 patients, with 61 experiencing recurrence (50.8%) and 59 remaining free of recurrence (49.2%). We assessed demographic factors, procedural parameters, comorbidities, and medication use to identify predictors of AF recurrence.

The results indicated that AF type was strongly linked to recurrence, with patients with persistent AF experiencing a significantly higher recurrence rate compared to those with paroxysmal AF (90.2% vs. 9.8%, *p* < 0.001). Additionally, rhythm control through electrical cardioversion was associated with increased recurrence (78.7% vs. 33.9% for non-recurrence, *p* < 0.001). Concerning drug treatments, the use of SGLT2i was connected to a lower recurrence of AF (24.6% in the SGLT2i group compared to 50.8% without, *p* = 0.003). The median age also showed a significant difference between the groups (69.5 years in those with recurrence versus 69 years in those without recurrence, *p* = 0.001), although the difference is clinically minor (Table 1).

Other demographic variables such as gender, cardiometabolic comorbidities (hypertension, diabetes mellitus, obesity, dyslipidaemia) and most drug treatments were not significantly associated with AF recurrence, although some (e.g., spironolactone or statins) showed trends that could be clinically relevant. Procedural parameters and radiation exposure dose varied between groups, but only radiation exposure time was marginally significant (*p* = 0.048) (Table 1).

Compared to patients without AF recurrence, those with recurrence showed a significantly larger left atrial diameter (44.5 mm vs. 42 mm, *p* = 0.039). Other parameters, including NT-proBNP, tricuspid regurgitation velocity (TRV), E/e’ ratio, interventricular septum (IVS), posterior wall (PW), left ventricular end-diastolic volume (LVEDV), left ventricular end-systolic volume (LVESV), relative wall thickness (RWT), and ejection fraction (EF), did not reach statistical significance. However, NT-proBNP levels were close to significance (685.5 vs. 541 pg/mL, *p* = 0.068), indicating a potential clinical trend (Table 2).

The analysis of antiarrhythmic treatment indicates that most patients who underwent electrical cardioversion received amiodarone (78.1%). Among these, the proportion of patients experiencing AF recurrence was higher (82.2%) compared to those without recurrence (68.4%), although this difference was not statistically significant (*p* = 0.685). Flecainide, propafenone, and sotalol were used less frequently, and the distribution of recurrence in these groups did not show significant differences. These findings suggest that, within the analysed cohort, the type of antiarrhythmic used in electrical cardioversion patients was not significantly associated with AF recurrence risk, although the trends observed for flecainide could be examined further in a larger sample (Table 3).

**Figure 1:**
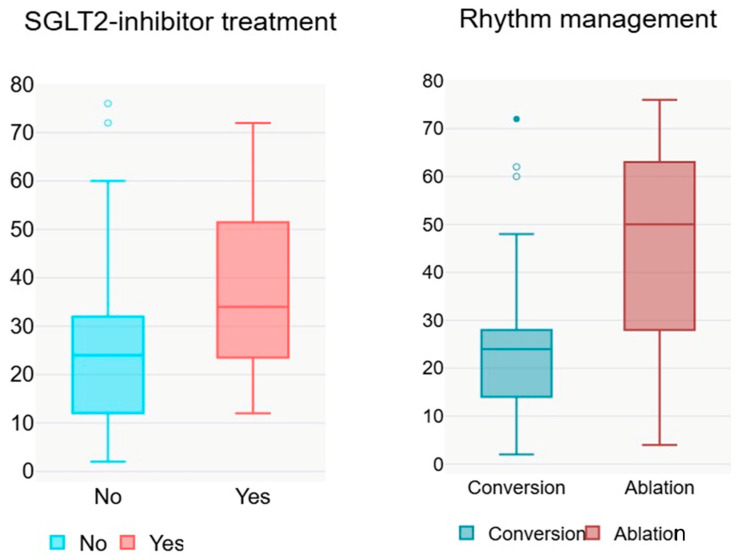
Boxplots illustrating the association between treatment strategies and time (weeks) to atrial fibrillation recurrence. Patients treated with SGLT2i (**left**) had a longer median time to recurrence compared with those without such therapy. Similarly, rhythm control with ablation (**right**) was associated with a substantially prolonged time to recurrence compared with electrical cardioversion.

Linear regression analysis for the time from conversion to AF recurrence revealed that certain variables were significantly associated with a longer interval. Rhythm control by catheter ablation was associated with a positive B of 18.245 (*p* = 0.001, 95% CI 7.559–28.930), indicating that patients treated with this intervention had a longer time to recurrence compared with those who underwent electrical cardioversion (Figure 1). Amiodarone administration was also significantly associated with a longer time to recurrence (B = 14.985, *p* = 0.008, 95% CI 3.996–25.974). The use of SGLT2i also showed a positive association (B = 12.143, *p* = 0.026, 95% CI 1.526–22.760), suggesting a protective effect on the time to AF recurrence. Specifically, in patients who received SGLT2i therapy, the median time to AF recurrence was 12 weeks longer (Table 4). In contrast, prior conversion and AF type (paroxysmal vs. persistent) were not significantly associated with time to recurrence, indicating that these variables did not notably influence the duration until AF recurrence in the analysed cohort.

Comparing patients with paroxysmal and persistent AF, significant differences were observed. Procedure time was longer in patients with persistent AF (150 vs. 95 min, *p* = 0.026). The left atrial size was larger in those with persistent AF (46 vs. 41.5 mm, *p* < 0.001), indicating more advanced structural remodelling associated with persistent forms. Additionally, the thickness of the posterior wall of the left ventricle was greater in persistent AF (11 vs. 9 mm, *p* = 0.043), and the NT-proBNP level was significantly higher (588 vs. 546 pg/mL, *p* = 0.001) (Table 5).

Patients undergoing electrical cardioversion had significantly higher values of left atrial diameter and NT-proBNP compared to those treated with cryoablation. In contrast, cryoablation was associated with more favourable echocardiographic parameters and lower NT-proBNP levels (Table 6). Procedural parameter values show that radiation dose did not differ significantly between groups (601 [316–1845] µGy/m^2^ for cryoablation versus 442 [215–734] µGy/m^2^ for RFA, *p* = 0.136), but radiation exposure time and total procedure duration were significantly longer in the radiofrequency group compared to cryoablation (19 [14–30] min versus 6 [4–10] min for radiation time, *p* < 0.001; 110 [80–150] min versus 160 [130–180] min for procedure duration, *p* < 0.001) (Table 6).

The median values and interquartile ranges for radiation dose, exposure time, procedure duration, echocardiographic parameters, and NT-proBNP show no significant differences between patients with and without cardioversion prior to catheter ablation, according to the reported *p* values. Although observations such as procedure time and left ventricular end-diastolic volume (LVEDV) tend to be lower in patients with prior cardioversion, these differences do not reach statistical significance. The results indicate that a history of prior cardioversion does not significantly affect radiation exposure, procedure duration, or the structural and functional parameters assessed by echocardiography in the study (Table 7).

Table 8 displays the procedural and echocardiographic characteristics of patients based on SGLT2i treatment. The median values and interquartile ranges indicate no significant differences between patients who received SGLT2i and those who did not, across all variables analysed, including radiation dose and duration, procedure length, and structural and functional cardiac parameters assessed by echocardiography, as well as NT-probnp. Although some values, such as PW and LVESV, exhibit slight trends between groups, these differences do not achieve statistical significance. The findings suggest that SGLT2i administration does not affect procedural or echocardiographic parameters within the context evaluated.

For the univariate survival analysis, the most relevant variables were selected: AF type, rhythm control therapy used, use of amiodarone, and administration of SGLT2i. Univariate Kaplan–Meier analysis was employed to assess differences in event-free survival between the levels of these variables, with the log-rank test (Mantel–Cox) used to compare survival distributions.

**Figure 2:**
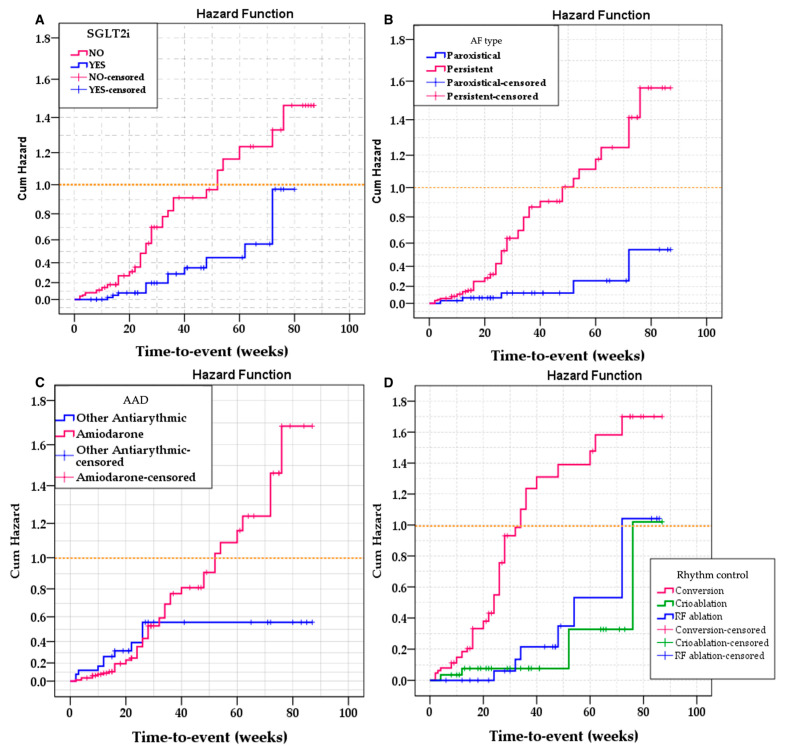
Kaplan–Meier survival analysis by clinical and therapeutic variables. Cumulative hazard curves are presented for: (**A**) SGLT2i use, (**B**) type of AF (paroxysmal versus persistent), (**C**) AAD (amiodarone versus other antiarrhythmics), and (**D**) rhythm control strategy (cardioversion, cryoablation, and RFA). Log-rank analysis demonstrated significant differences for AF type, cardioversion method, and SGLT2i use, but not for amiodarone use. SGLT2i, sodium–glucose cotransporter 2 inhibitors; AF, atrial fibrillation; AAD, antiarrhythmic drugs, RF, radiofrequency.

The results demonstrated significant differences in event-free survival based on AF type (Log Rank = 11.242; df = 1; *p* = 0.001), rhythm control therapy used (Log Rank = 21.879; df = 2; *p* < 0.001), and administration of SGLT2i (Log Rank = 7.869; df = 1; *p* = 0.005). Conversely, the use of amiodarone was not significantly linked to variations in survival (Log Rank = 1.186; df = 1; *p* = 0.276) (Figure 2).

For the multivariable analysis, we included the variables: type of procedure (RFA, cryoablation, electrical cardioversion—reference), treatment with SGLT2i, age, NT-proBNP, AF type, and left atrial size. AS and NT-proBNP were incorporated to account for differences, as patients who required electrical cardioversion had significantly higher levels of both NT-proBNP and left atrial size compared to the rest of the group (Table 9).

Results showed that both cryoablation (HR = 0.226, 95% CI: 0.089–0.573, *p* = 0.002) and radiofrequency ablation (HR = 0.293, 95% CI: 0.131–0.654, *p* = 0.003) were linked with a significantly lower risk of event compared to conversion (Table 9). SGLT2i treatment was also associated with a decreased risk of recurrence (HR = 0.421, 95% CI: 0.266–0.786, *p* = 0.007). Age was an independent predictor, with each extra year increasing the risk by around 4% (HR = 1.04, 95% CI: 1.002–1.07, *p* = 0.038). Conversely, NT-proBNP and left atrial size had no significant independent effect on risk after adjustment (*p* > 0.05), and the AF type was not significantly linked to risk (HR = 1.77, 95% CI: 0.60–5.20, *p* = 0.299).

**Figure 3:**
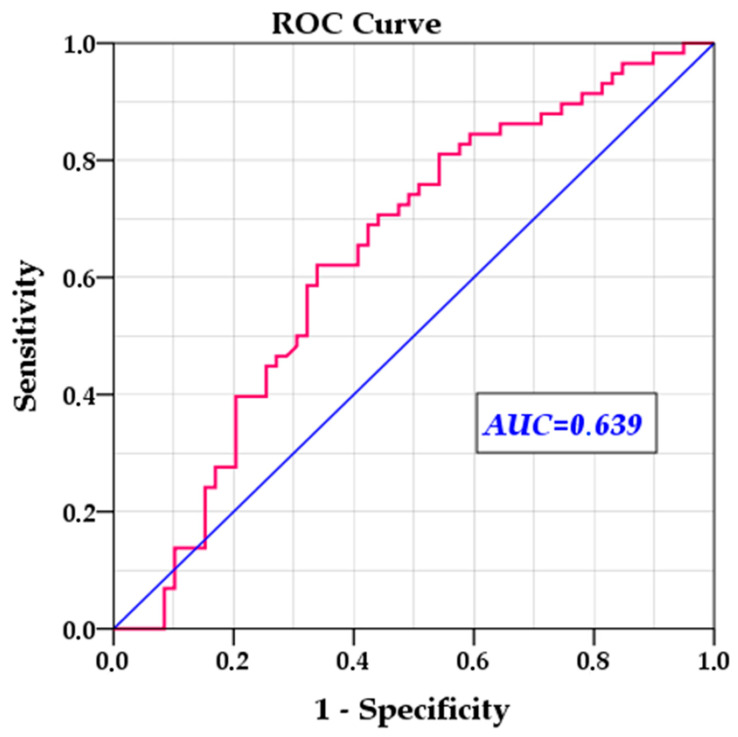
ROC curve for the optimised multivariable Cox model, using hazard probability for predicting relapse risk. AUC = 0.639 (*p* = 0.010, 95% CI: 0.537–0.741). AUC, area under the curve.

To validate and verify the efficiency of the multivariable model, we removed statistically insignificant variables and reran the multivariable Cox regression, this time calculating the hazard probability for each individual case. We then used this probability to evaluate the model’s ability to predict relapse risk. The ROC analysis showed an AUC of 0.639 (SE = 0.052, *p* = 0.010, 95% CI: 0.537–0.741), indicating moderate predictive power, significantly different from chance (Figure 3).

## 4. Discussion

The present study demonstrates that, in patients with HFpEF and concomitant AF, catheter ablation—whether by RFA or cryoablation—provides significantly more durable rhythm control than electrical cardioversion, reducing AF recurrence and prolonging arrhythmia-free survival. Importantly, adjunctive therapy with SGLT2i independently contributes to improved rhythm stability, extending the AF-free period by approximately 12 weeks, irrespective of procedural choice or baseline atrial and biomarker characteristics. These findings highlight a synergistic strategy in HFpEF, whereby targeted substrate modification via ablation combined with metabolic and electrophysiologic modulation through SGLT2 inhibition offers superior maintenance of sinus rhythm, addressing a critical gap in the management of this high-risk population.

Catheter ablation is a first-line treatment option for AF in selected patients, and randomised trials consistently demonstrate its superiority over drug therapy for symptom and rhythm control, with prognostic benefits shown in HFrEF. Yet, whether these advantages apply to HFpEF remains uncertain [12]. HFpEF is a distinct syndrome characterised by multimorbidity, atrial and ventricular remodelling, and elevated filling pressures [13], features that shape the atrial substrate and may alter both the effectiveness and the clinical outcomes of rhythm strategies. AF is highly prevalent in HFpEF and often leads to decompensation, hospitalisation, and reduced quality of life, but guidance on the optimal rhythm strategy for this phenotype is limited [12]. We therefore concentrated specifically on patients with HFpEF and AF to address a practical gap: how electrical cardioversion compares with modern ablation techniques, and whether adjunctive haemodynamic- and metabolism-modifying therapies, such as SGLT2i, influence rhythm stability in routine care.

In the present study, we showed that catheter ablation surpasses electrical cardioversion in maintaining sinus rhythm and extending arrhythmia-free survival in patients with HFpEF and AF, an advantage which persists after accounting for age, atrial size, natriuretic peptides, and AF type, suggesting that the effect is not due to baseline differences. Mechanistically, the result is plausible in HFpEF. These patients often have atrial myopathy caused by pressure/volume overload, fibrosis, microvascular dysfunction, and inflammation [14]. Cardioversion temporarily restores rhythm but leaves the substrate unchanged, making relapse likely when filling pressures fluctuate or triggers re-emerge. Ablation, on the other hand, targets triggers and alters the substrate, decreasing the chance that the same anatomic-electrophysiologic circuits will restart AF. In a phenotype characterised by atrial stiffness and remodelling, modifying the substrate becomes especially important.

Recent studies showed similar results, yet data in speciality literature remains inconclusive. One study demonstrated that, in HFpEF patients with AF, catheter ablation provides clear physiological and symptomatic benefits, lowering peak exercise PCWP, increasing peak VO_2_, and reducing NT-proBNP, with 50% of patients no longer fulfilling invasive exercise criteria for HFpEF at 6 months post-procedure [15]. However, in a meta-analysis that pooled 12 randomised clinical trials, benefits on major endpoints seem limited for HFpEF: ablation reduced HF events and cardiovascular mortality in HFrEF but not in HFpEF [16]. A recent CABANA analysis using a modified H_2_FPEF score indicates that HF phenotype is significant: patients with a high likelihood of HFpEF experienced a lower risk of cardiovascular hospitalisation or death with catheter ablation and saw the greatest reduction in AF recurrence, with consistent results in an echo-defined HFpEF subset [9]. Real-world data reflect symptom and utilisation improvements despite increased rhythm vulnerability: in a 7020-patient registry, HFpEF had the highest 3-year AF recurrence (53%), yet catheter ablation still reduced AF-related hospitalisations and improved AF symptom and burden scores across all HF subtypes [17]. Taken together, our findings and the contemporary (though mixed) literature support catheter ablation as a trigger and substrate-modifying therapy, as the preferred rhythm-control strategy over electrical cardioversion for appropriately selected HFpEF patients with AF, resulting in more durable sinus rhythm and meaningful clinical improvements, while effects on other major outcomes remain uncertain and require confirmation in larger randomised trials. Future studies could also explore phenotype-guided AF ablation in HFpEF to assess whether customising lesion sets based on specific clinical phenotypes enhances rhythm outcomes.

A second key finding of our study is that SGLT2i therapy is linked to a lower AF recurrence rate and a longer arrhythmia-free period in HFpEF patients, regardless of procedural choice and baseline clinical characteristics. Additionally, the effect size is clinically meaningful: SGLT2i use is associated with approximately a 12-week longer AF-free period after rhythm restoration. For patients and clinicians, an extra three months in sinus rhythm can result in fewer early re-presentations, reduced need for repeat cardioversion or antiarrhythmic escalation, and more time to optimise upstream HFpEF care.

Across contemporary datasets, SGLT2i treatment consistently correlates with improved rhythm stability in AF and seems to complement procedural strategies: after catheter ablation, their use is associated with fewer recurrences and downstream interventions (such as cardioversion, antiarrhythmic escalation, or re-do ablation), as well as longer AF-free survival, endorsing a practical “ablation-plus-SGLT2i” approach rather than treating these options as alternatives [11,18]. Beyond the post-ablation setting, trial-level syntheses indicate a broader anti-arrhythmic signal, with SGLT2i users experiencing less atrial arrhythmia overall and lower sudden cardiac death rates, albeit with phenotype nuances: benefits are most evident in HFrEF and not consistently seen in HFpEF, a distinction that helps explain heterogeneity across studies and highlights the need for HFpEF-specific trials [19,20]. Complementing these meta-analytic data, pooled analyses across randomised cohorts further support a reduction in incident atrial arrhythmia with SGLT2i compared to placebo, reinforcing that the rhythm effect is not confined to any one SGLT2i molecule or trial programme, so the effect appears general to the class [21]. Beyond the observed benefits on AF recurrence, SGLT2i have recently been linked to a lower risk of sudden cardiac death in patients with type 2 diabetes, HF, or CKD in adjudicated randomised evidence (meta-analysis of 8 RCTs; OR 0.82, 95% CI 0.72–0.94) [22], a finding that may also reflect class-related antiarrhythmic effects. Recent studies have also assessed the combined effect of SGLT2i and glucagon-like peptide-1 receptor agonists (GLP-1RA) on AF recurrence after catheter ablation. Combined SGLT2i and GLP-1RA therapy was linked to a 56% reduction in 1-year AF recurrence after catheter ablation in diabetic patients with paroxysmal AF, indicating cardioprotective effects beyond glucose lowering [23]. Mechanistically, converging basic and translational evidence indicates that the antiarrhythmic effects of SGLT2i result from a dual action on both arrhythmic triggers and substrates [24]. SGLT2i reduce intracellular sodium by inhibiting the cardiac Na^+^/H^+^ exchanger and decreasing late INa, which limits Ca^2+^ overload, calms CaMKII activity, stabilises RyR2 and the Na^+^/Ca^2+^ exchanger, and consequently lowers EAD/DAD triggers. Simultaneously, they promote a “fasting-like” metabolic and autophagic state in the myocardium (via AMPK/SIRT1/HIF pathways), reducing oxidative stress, decreasing NLRP3-driven inflammation, and preventing structural remodelling such as fibrosis and gap-junction malfunction [25]. These combined effects enhance substrate and conduction stability, which are crucial for AF maintenance and the durability of post-ablation outcomes [24]. In summary, our findings support a practical, integrated approach to managing rhythm in HFpEF: prioritising catheter ablation combined with SGLT2i to enhance rhythm stability. The underlying reasoning for using SGLT2i, which reduces triggers and stabilises the cardiac substrate, corresponds with the observed longer periods without AF and fewer recurrences, providing clear clinical benefits. While these results are relevant for current practice, as this analysis is observational and residual confounding cannot be excluded, they remain hypothesis-generating. Future HFpEF-specific, prospective trials with standardised monitoring are needed to determine the optimal timing (pre-ablation loading vs. post-ablation maintenance strategies), measure effects on AF burden, symptoms, HF hospitalisation, and safety.

In our multivariable analysis, older age remains an independent predictor of AF recurrence in HFpEF even after adjusting for AF type, LA size, NT-proBNP, and the selected rhythm-control strategy. This indicates that age reflects aspects of atrial vulnerability not fully captured by routine structural or biomarker measurements. Age remains a consistent and independent predictor of AF recurrence across various studies involving both non-surgical and surgical ablation procedures: patients over the age of 70 exhibit notably higher relapse rates post-ablation, a phenomenon likely attributable to age-related atrial remodelling, which includes dilatation, conduction slowing, extended refractory periods, fibrosis, and sinus node dysfunction [26]. This remodelling is often compounded by comorbid conditions that further elevate the risk of recurrence [26]. These observations have practical implications. Firstly, they advocate for early, proactive rhythm control in suitable HFpEF patients before age-related substrate changes become entrenched. Secondly, they support stricter upstream optimisation in older patients: congestion control, blood pressure and weight management, and careful rhythm follow-up, to reduce trigger load and stabilise the substrate after restoring sinus rhythm. Lastly, shared decision-making should explicitly consider age-related relapse risk when choosing between cardioversion and ablation, planning surveillance intensity, and setting expectations for rhythm durability.

Our data indicate that, in HFpEF with AF, cryoablation and RFA generally produce similar rhythm outcomes, consistent with current understanding that effectiveness depends as much on substrate as on the energy method used. In our cohort, they differ mainly in procedural aspects: RFA procedures tend to take longer overall, while cryoablation results in shorter total procedure durations. However, cryoablation involves longer fluoroscopy times, although the radiation dose is comparable for both. These differences have practical implications in HFpEF, where reducing invasiveness and hemodynamic stress is important, but considerations like radiation exposure and lab workflow efficiency are also relevant. Shorter procedures may lead to less sedation time and quicker recovery for older, comorbid patients. Lower fluoroscopy exposure might be prioritised in centres utilising advanced mapping workflows. Given the non-randomised design and operator-dependent technique, the choice between cryoablation and RFA should remain personalised, guided by anatomy, AF pattern, centre expertise, and logistical considerations [27], while our data reassure that, from a rhythm-control perspective, either modality is a reasonable cornerstone for HFpEF when ablation is pursued.

### Study Limitations

This study was conducted at a single centre utilising a retrospective, observational design. Such characteristics inherently limit broad generalisability, as practice patterns, referral pathways, and patient characteristics may vary in different settings. Additionally, they imply that operator technique and patient-specific clinical decisions were not standardised across all cases, which may introduce variability in procedural approaches and post-procedural care. Although this constrains precise control over every factor, it accurately reflects real-world clinical practice and provides a pragmatic perspective on outcomes within routine healthcare environments.

The study population was deliberately diverse, including both paroxysmal and persistent atrial fibrillation, reflecting the range encountered in routine clinical practice. Most patients were treated with antiarrhythmic medication for several weeks prior to the intervention and continued therapy during the post-procedure blanking period. This approach aligns with standard management and supports rhythm stabilisation during the early recovery phase. Importantly, antiarrhythmic drug use during the blanking period is generally understood to affect early recurrences rather than long-term rhythm outcomes, so any impact on our primary endpoints beyond the blanking period is likely limited.

Finally, the sample size, while adequate for identifying multiple clinically significant associations, limits the precision of subgroup analyses and the capacity to examine subtle distinctions between paroxysmal and persistent AF. Collectively, these considerations are manageable and characteristic of targeted, real-world investigations but should be considered when generalising the findings to larger or differently constructed populations.

Future large, prospective, multicentre studies with standardised follow-up would be valuable to validate these findings, enhance the accuracy of effect estimates, and guide patient selection.

## 5. Conclusions

For HFpEF patients with AF, catheter ablation (cryoablation and RFA) provides more durable rhythm control than electrical cardioversion, with a significantly lower risk of AF recurrence rate and increased time until recurrence, supporting its role as a preferred rhythm-control strategy in carefully selected HFpEF patients. SGLT2i, which have recently emerged as a promising mechanistically driven therapy in AF, appear to confer benefits in reducing AF recurrence and improving rhythm stability. Focusing on a HFpEF population and jointly assessing the procedural approach and SGLT2i therapy provide timely, novel insights that are relevant to everyday practice. Although further prospective studies are necessary to confirm these findings, our results endorse an integrated, phenotype-specific approach to rhythm management in HFpEF.

## Figures and Tables

**Table 1:** Clinical and demographic characteristics of patients with AF and their association with AF recurrence.

Variable		Total (%)	Non-Recurrent AF	Recurrent AF	*p*
			59 (49.2%)	61 (50.8%)	
***Demographic data***					
**Sex**					
***Female***		52 (43.3%)	26 (44.1%)	26 (42.6%)	0.873
***Male***			33 (55.9%)	35 (57.4%)	
**Age (yrs)**			69 (67–70)	69.5 (65–76)	0.001
***Pathological and procedural parameters***					
**AF type**					
***Paroxysmal***		32 (26.7%)	26 (44.1%)	6 (9.8%)	<0.001
***Persistent***		88 (73.3%)	33 (55.9%)	55 (90.2%)	
**Rhythm control**					
***Cardioversion***		68 (56.7%)	20 (33.9%)	48 (78.7%)	<0.0018
***Cryoablation***		29 (24.2%)	24 (40.7%)	5 (8.2%)	
***RFA***		23 (19.2%)	15 (25.4%)	8 (13.1%)	
**Rhythm control (generic)**					
***Cardioversion***			20 (33.9%)	48 (78.7%)	<0.001
***Catheter ablation***			39 (66.1%)	13 (21.3%)	
**Radiation exposure dose (μGy/m^2^)**			463.4 (180.2–600.6)	699.2 (216.1–885.9)	0.301
**Radiation exposure time (min)**			11.20 (10–25.10)	8.25 (3.90–16.50)	0.048
**Procedure time (min)**			150 (80–180)	140 (130–180)	0.371
**Amiodarone**		81 (75.7%)	35 (68.6%)	46 (82.1%)	0.104
***Comorbidities***					
**Mitral regurgitation**	None	10 (8.3%)	6 (10.2%)	4 (6.6%)	0.432
	Mild	66 (55.0%)	35 (59.3%)	31 (50.8%)	
	Moderate	43 (35.8%)	18 (30.5%)	25 (41.0%)	
	Severe	1 (0.8%)	0 (0.0%)	1 (1.6%)	
**Tricuspid regurgitation**	None	23 (19.2%)	14 (23.7%)	9 (14.8%)	0.624
	Mild	46 (38.3%)	22 (37.3%)	24 (39.3%)	
	Moderate	41 (34.2%)	19 (32.2%)	22 (36.1%)	
	Severe	10 (8.3%)	4 (6.8%)	6 (9.8%)	
**Aortic regurgitation**	None	84 (69.7%)	42 (70.7%)	42 (68.9%)	0.413
	Mild	31 (26.1%)	16 (27.6%)	15 (24.6%)	
	Moderate	5 (4.2%)	1 (1.7%)	4 (6.6%)	
**Arterial hypertension**		106 (88.3%)	51 (86.4%)	55 (90.2%)	0.525
**Diabetes mellitus**		36 (30.3%)	18 (30.5%)	18 (30%)	0.952
**CCS**		21 (17.6%)	11 (19.0%)	10 (16.4%)	0.713
**Overweight/obesity**		62 (51.7%)	35 (59.3%)	27 (44.3%)	0.099
**CKD**		12 (10.0%)	8 (13.6%)	4 (6.6%)	0.201
**Hypothyroidism**		20 (16.8%)	9 (15.3%)	11 (18.3%)	0.653
**Dyslipidemia**		66 (55.0%)	34 (57.6%)	32 (52.5%)	0.568
**COPD**		11 (9.2%)	3 (5.1%)	8 (13.1%)	0.128
**OSA**		18 (15.0%)	10 (16.9%)	8 (13.1%)	0.556
**Anaemia**		9 (7.5%)	3 (5.1%)	6 (9.8%)	0.323
***Treatment***					
**Beta-blockers**		111 (92.5%)	56 (94.9%)	55 (90.2%)	0.323
**CaCB**		30 (25.0%)	15 (25.4%)	15 (24.6%)	0.916
**Antiplatelets**		1 (0.8%)	0 (0.0%)	1 (1.6%)	0.323
**VKAs**		5 (4.2%)	1 (1.7%)	4 (6.6%)	0.183
**DOACs**		115 (95.8%)	58 (98.3%)	57 (93.4%)	0.183
**ACEI/ARB**		91 (75.8%)	46 (78.0%)	45 (73.8%)	0.591
**ARNI**		4 (3.3%)	1 (1.7%)	3 (4.9%)	0.325
**Spironolactone**		41 (34.2%)	17 (28.8%)	24 (39.3%)	0.224
**SGLT2i**		45 (37.5%)	30 (50.8%)	15 (24.6%)	0.003
**Digoxin**		2 (1.7%)	0 (0.0%)	2 (3.3%)	0.161
**Diuretics**		76 (63.3%)	37 (62.7%)	39 (63.9%)	0.889
**Statins**		77 (64.2%)	42 (71.2%)	35 (57.4%)	0.115
**Ezetimibe**		5 (4.2%)	2 (3.4%)	3 (4.9%)	0.675

AF, atrial fibrillation; RFA, radiofrequency ablation; CCS, chronic coronary syndrome; CKD, chronic kidney disease; COPD, chronic obstructive pulmonary disease; OSA, obstructive sleep apnoea; CaCB, calcium channel blockers; VKA, vitamin K antagonists; DOACs, direct oral anticoagulants; ACEI, angiotensin-converting enzyme inhibitors; ARB, angiotensin receptor blockers; ARNI, angiotensin receptor-neprilysin inhibitor; SGLT2i, sodium–glucose cotransporter 2 inhibitors.

**Table 2:** Echocardiographic parameters and cardiac biomarkers according to AF recurrence.

Variable	Non-Recurrent AF	Recurrent AF	*p*
**LA (mm)**	42 (41–46)	44.5 (42–47)	0.039
**E/e’ ratio**	12 (8–13)	7.33 (6.25–10)	0.833
**TRV (m/s)**	2.80 (2.40–3.20)	2.91 (2.30–3.20)	0.836
**IVS (mm)**	11 (10–12)	11 (10–11)	0.851
**PW (mm)**	11 (9–11)	10 (10–11)	0.922
**LVEDV (mm)**	48 (45–51)	50.5 (46–53)	0.551
**LVESV (mm)**	34 (32–36)	35.5 (28–38)	0.903
**RWT**	0.44 (0.38–0.47)	0.4116 (0.38–0.43)	0.611
**EF (%)**	54 (50–55)	55 (55–59)	0.775
**NT-pro BNP (pg/mL)**	541 (346–705)	685.5 (588–1444)	0.068

AF, atrial fibrillation; LA, left atrium; TRV, tricuspid regurgitation velocity at rest; IVS, interventricular septum; PW, posterior wall; LVEDV, left ventricular end-diastolic volume; LVESV, left ventricular end-systolic volume; RWT, relative wall thickness; EF, ejection fraction.

**Table 3:** Distribution of antiarrhythmic treatment in patients who underwent electrical cardioversion and its association with AF recurrence.

AAD	Total (%)	Non-Recurrent AF	Recurrent AF	*p*
**Amiodarone**	50 (78.1%)	13 (68.4%)	37 (82.2%)	0.685
**Flecainide**	8 (12.5%)	5 (26.3%)	3 (6.7%)	0.194
**Propafenone**	5 (7.8%)	1 (5.3%)	4 (8.9%)	0.998
**Sotalol**	1 (1.6%)	0 (0.0%)	1 (2.2%)	0.993
**Total**	64 (100%)	19 (100%)	45 (100%)	

AF, atrial fibrillation; AAD, antiarrhythmic drugs.

**Table 4:** Multiple linear regression analysis for factors associated with time to AF recurrence.

Variable	B-Coefficient	*p*	95% CI
**Prior cardioversion**	−0.678	0.950	−22.372–21.016
**AF type**	3.223	0.705	−13.745–20.190
**Treatment by catheter ablation**	18.245	0.001	7.559–28.930
**Amiodarone treatment**	14.985	0.008	3.996–25.974
**SGLT2i treatment**	12.143	0.026	1.526–22.760

AF, atrial fibrillation; CI, confidence interval; SGLT2i, sodium–glucose cotransporter 2 inhibitors.

**Table 5:** Procedural and echocardiographic characteristics of patients with paroxysmal versus persistent AF.

Variable	Paroxysmal AF	Persistent AF	*p*
**Radiation dose (µGy/m^2^)**	743.27 (532.06–1165.00)	337.39 (175.20–688.42)	0.253
**Radiation exposure time (min)**	23.00 (18.70–41.45)	10.00 (6.50–10.75)	0.153
**Procedure time (min)**	95.00 (85.00–170.00)	150.00 (130.00–180.00)	0.026
**LA (mm)**	41.50 (40.50–42.00)	46.00 (42.50–47.00)	<0.001
**E/e’ ratio**	12.47 (11.00–13.47)	8.00 (7.23–11.00)	0.828
**TRV (m/s)**	3.20 (2.99–3.35)	2.65 (2.30–3.08)	0.431
**IVS (mm)**	11.50 (10.50–12.50)	11.00 (10.00–11.00)	0.377
**PW (mm)**	9.00 (8.00–10.50)	11.00 (10.00–11.00)	0.043
**LVEDV (mm)**	50.00 (47.50–51.50)	48.00 (45.50–52.00)	0.192
**LVESV (mm)**	34.00 (33.00–36.00)	34.00 (30.00–36.50)	0.100
**RWT**	0.3556 (0.3201–0.4314)	0.4348 (0.4116–0.4744)	0.106
**EF (%)**	57.50 (54.50–60.50)	55.00 (50.00–55.00)	0.203
**NT-pro BNP (pg/mL)**	546.50 (317.00–676.50)	588.00 (445.00–1232.00)	0.001

AF, atrial fibrillation; LA, left atrium; TRV, tricuspid regurgitation velocity at rest; IVS, interventricular septum; PW, posterior wall; LVEDV, left ventricular end-diastolic volume; LVESV, left ventricular end-systolic volume; RWT, relative wall thickness; EF, ejection fraction.

**Table 6:** Procedural and echocardiographic characteristics of patients according to rhythm management strategy.

Variable	Cardioversion	Cryoablation	RFA	*p*
Radiation dose (µGy/m^2^)	Not applicable	601 (316–1845)	442 (215–734)	0.136
Radiation exposure time (min)	Not applicable	19 (14–30	6 (4–10)	<0.001
Procedure time (min)	Not applicable	110 (80–150)	160 (130–180)	<0.001
LA (mm)	45 (44–48) *	42 (42–44) *	46 (42–47)	0.004
E/e’ ratio	10 (8–12)	10 (9–12)	8 (6–13)	0.280
TRV (m/s)	2.6 (2.5–3.0)	2.8 (2.6–3.2)	2.8 (2.2–3.4)	0.694
IVS (mm)	11 (10–12)	12 (11–12)	11 (10–11)	0.675
PW (mm)	10 (10–12)	10 (9–11)	11 (10–11)	0.713
LVEDV (mm)	48 (44–52)	51 (48–52)	47 (46–51)	0.663
LVESV (mm)	33 (30–36)	36 (34–37)	31 (29–36)	0.975
RWT	0.44 (0.39–0.50)	0.39 (0.36–0.46)	0.43 (0.41–0.48)	0.378
EF (%)	50 (50–55)	55 (55–61)	55 (50–55)	0.593
NT-proBNP (pg/mL)	959 (714–1605) *	541 (317–677) *	656 (448–1485)	0.013

RFA, radiofrequency ablation; LA, left atrium; TRV, tricuspid regurgitation velocity at rest; IVS, interventricular septum; PW, posterior wall; LVEDV, left ventricular end-diastolic volume; LVESV, left ventricular end-systolic volume; RWT, relative wall thickness; EF, ejection fraction. * yellow highlighting shows significant pairwise differences.

**Table 7:** Procedural and echocardiographic characteristics of patients with/without electrical cardioversion prior to catheter ablation.

Variable	No Prior Cardioversion	Prior Cardioversion	*p*
Radiation dose (µGy/m^2^)	601 (463–850)	198 (170–549)	0.541
Radiation exposure time (min)	19 (10–25)	9 (4–11)	0.296
Procedure time (min)	180 (150–180)	115 (60–130)	0.083
LA (mm)	43 (41–46)	44 (42–49)	0.937
E/e’ ratio	12 (8–13)	9 (8–10)	0.743
TRV (m/s)	3.16 (2.80–3.49)	2.35 (2.30–2.65)	0.894
IVS (mm)	11 (10–11)	11 (10–12)	0.145
PW (mm)	11 (9–11)	10 (10–11)	0.412
LVEDV (mm)	48 (46–49)	53 (46–55)	0.085
LVESV (mm)	32 (30–34)	37 (36–38)	0.575
RWT	0.47 (0.41–0.48)	0.41 (0.38–0.43)	1.000
EF (%)	54 (50–55)	57 (55–60)	0.603
NT-proBNP (pg/mL)	705 (445–1444)	565 (349–648)	0.191

LA, left atrium; TRV, tricuspid regurgitation velocity at rest; IVS, interventricular septum; PW, posterior wall; LVEDV, left ventricular end-diastolic volume; LVESV, left ventricular end-systolic volume; RWT, relative wall thickness; EF, ejection fraction.

**Table 8:** Procedural and echocardiographic characteristics of patients with/without SGLT2i treatment.

Variable	Without SGLT2i	With SGLT2i	*p*
Radiation dose (µGy/m^2^)	539 (277–743)	463 (154–839)	0.620
Radiation exposure time (min)	14 (9–35)	10 (7–14)	0.714
Procedure time (min)	155 (90–180)	150 (110–165)	0.405
LA (mm)	44 (42–46)	43 (40–47)	0.431
E/e’ ratio	11 (8–13)	8 (7–11)	0.589
TRV (m/s)	3.00 (2.47–3.35)	2.78 (2.35–3.08)	0.301
IVS (mm)	11 (10–12)	11 (11–12)	0.134
PW (mm)	10 (10–11)	11 (10–11)	0.056
LVEDV (mm)	49 (46–52)	49 (47–52)	0.122
LVESV (mm)	36 (33–38)	32 (29–34)	0.144
RWT	0.43 (0.40–0.46)	0.41 (0.37–0.48)	0.542
EF (%)	55 (55–61)	54 (50–55)	0.139
NT-proBNP (pg/mL)	618 (493–714)	546 (269–1485)	0.608

SGLT2i, sodium–glucose cotransporter 2 inhibitors; LA, left atrium; TRV, tricuspid regurgitation velocity at rest; IVS, interventricular septum; PW, posterior wall; LVEDV, left ventricular end-diastolic volume; LVESV, left ventricular end-systolic volume; RWT, relative wall thickness; EF, ejection fraction.

**Table 9:** Multivariable Cox regression of variables that predict AF recurrence risk.

Variable	B-Coefficient	*p*	HR	CI 95%
**Cardioversion (ref.)**		<0.001	1.000	
**Cryoablation**	−1.487	0.002	0.226	0.089–0.573
**RFA**	−1.227	0.003	0.293	0.131–0.654
**SGLT2i**	−0.824	0.007	0.421	0.266–0.786
**Age**	0.037	0.038	1.038	1.002–1.074
**NT-proBNP**	<0.001	0.270	1.000	1.000–1.000
**AF type**	0.572	0.299	1.770	0.603–5.197
**LA diameter**	–0.016	0.577	0.984	0.938–1.040

HR, hazard ratio; CI, confidence interval; RFA, radiofrequency ablation; SGLT2i, sodium–glucose cotransporter 2 inhibitors; AF, atrial fibrillation; LA, left atrium.

## Data Availability

The raw data supporting the conclusions of this article will be made available by the authors upon reasonable request.

## Abbreviations

The following abbreviations are used in this manuscript:

HFpEF	heart failure with preserved ejection fraction
HF	heart failure
ESC	European Society of Cardiology
SGLT2i	sodium–glucose cotransporter 2 inhibitors
AF	Atrial fibrillation
HFrEF	heart failure with reduced ejection fraction
RFA	radiofrequency ablation
CCS	chronic coronary syndrome
CKD	chronic kidney disease
COPD	chronic obstructive pulmonary disease
OSA	obstructive sleep apnoea
CaCB	calcium channel blockers
VKA	vitamin K antagonists
DOACs	direct oral anticoagulants
ACEI	angiotensin-converting enzyme inhibitors
ARB	angiotensin receptor blockers
ARNI	angiotensin receptor-neprilysin inhibitor
LA	left atrium
TRV	tricuspid regurgitation velocity at rest
IVS	interventricular septum
PW	posterior wall
LVEDV	left ventricular end-diastolic volume
LVESV	left ventricular end-systolic volume
RWT	relative wall thickness
EF	ejection fraction
AAD	antiarrhythmic drugs
INa	late sodium channel current
CaMKII	calmodulin-dependent kinase II
RyR2	ryanodine receptor 2
EAD	early afterdepolarization
DAD	delayed afterdepolarization
AMPK	AMP-activated protein kinase
SIRT1	sirtuin 1
HIF	hypoxia-inducible factor
NLRP3	nuclear-binding domain-like receptor 3
GLP-1RA	glucagon-like peptide-1 receptor agonist
